# Epididymal leiomyosarcoma: Report of a rare case

**DOI:** 10.1002/ccr3.5511

**Published:** 2022-03-01

**Authors:** Mansoureh Dehghani, Mona Ariamanesh, Ali Khakbaz

**Affiliations:** ^1^ 461252 Radiation Oncologist Neyshabur University of Medical Sciences Neyshabur Iran; ^2^ 461252 Anatomical and Clinical Pathologist Department of Pathology Neyshabur University of Medical Sciences Neyshabur Iran; ^3^ 461252 Department of Urology Neyshabur University of Medical Sciences Neyshabur Iran

**Keywords:** epididymis, leiomyosarcoma, scrotal tumor

## Abstract

Epididymal leiomyosarcoma (LMS) is a rare malignancy. Because the risk of recurrence is high, proper approach is important. Here, we present a patient with scrotal swelling who underwent surgical excision via scrotal incision, and the histopathological diagnosis was epididymal LMS. The decision was then made to perform inguinal radical orchiectomy.

## Graphical abstract

Epididymal leiomyosarcoma is a rare but important differential diagnosis of a scrotal mass. Radical orchiectomy with high ligation of the spermatic cord is the preferred approach. Considering the risk of loco‐regional recurrence, further studies to establish a standard adjuvant approach are needed.

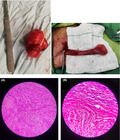

## 1 INTRODUCTION

Sarcomas of the genitourinary tract are rare and account for only 1%–2% of genitourinary malignancies.^1^ According to a large case series from the Memorial Sloan‐Kettering Cancer Center, 2.1% of soft tissue sarcomas arise in the genitourinary tract, of which 44% are para testicular, and leiomyosarcoma (LMS) is the most common type of para testicular sarcoma.^2^ There are no specific clinical findings to distinguish the type or the difference between this pathology and a benign tumor.^3^

The para testicular region consists of varying structures such as the epididymis, spermatic cord, tunica vaginalis, and fat‐ligament‐muscle supporting tissues.^4^ LMS is thought to arise from para testicular smooth muscle tissues, and they are prone to direct invasion and also early hematogenous spread. No standard treatment protocol is so far established; however, consensus suggests inguinal radical orchiectomy and high cord ligation. Some case series advocate the utilization of adjuvant radiotherapy.^5^

Here, we report a 60‐year‐old patient who presented with right scrotal founding and was found to have a leiomyosarcoma on excisional biopsy.

## 2 CASE

A 60‐year‐old male patient presented to the urology clinic with a complaint of right scrotal swelling without pain. The patient had noticed the swelling about 6 months earlier. There was no medical history other than type 2 diabetes controlled by oral medication. At physical examination, a round, firm mobile scrotal mass measuring about 5 × 5 cm was detected. No lymph node enlargement was detected, and the left testis and scrotum were normal. Ultrasound examination of the scrotum was performed which revealed a rather well‐defined lobulated, hypoechoic mass lesion. Tumor markers beta‐human chorionic gonadotropin (BHCG), lactate dehydrogenase (LDH), and alpha‐fetoprotein (AFP) were normal. Whole body CT‐scan (thorax, abdomen and pelvic) showed no signs of metastasis.

**FIGURE 1:**
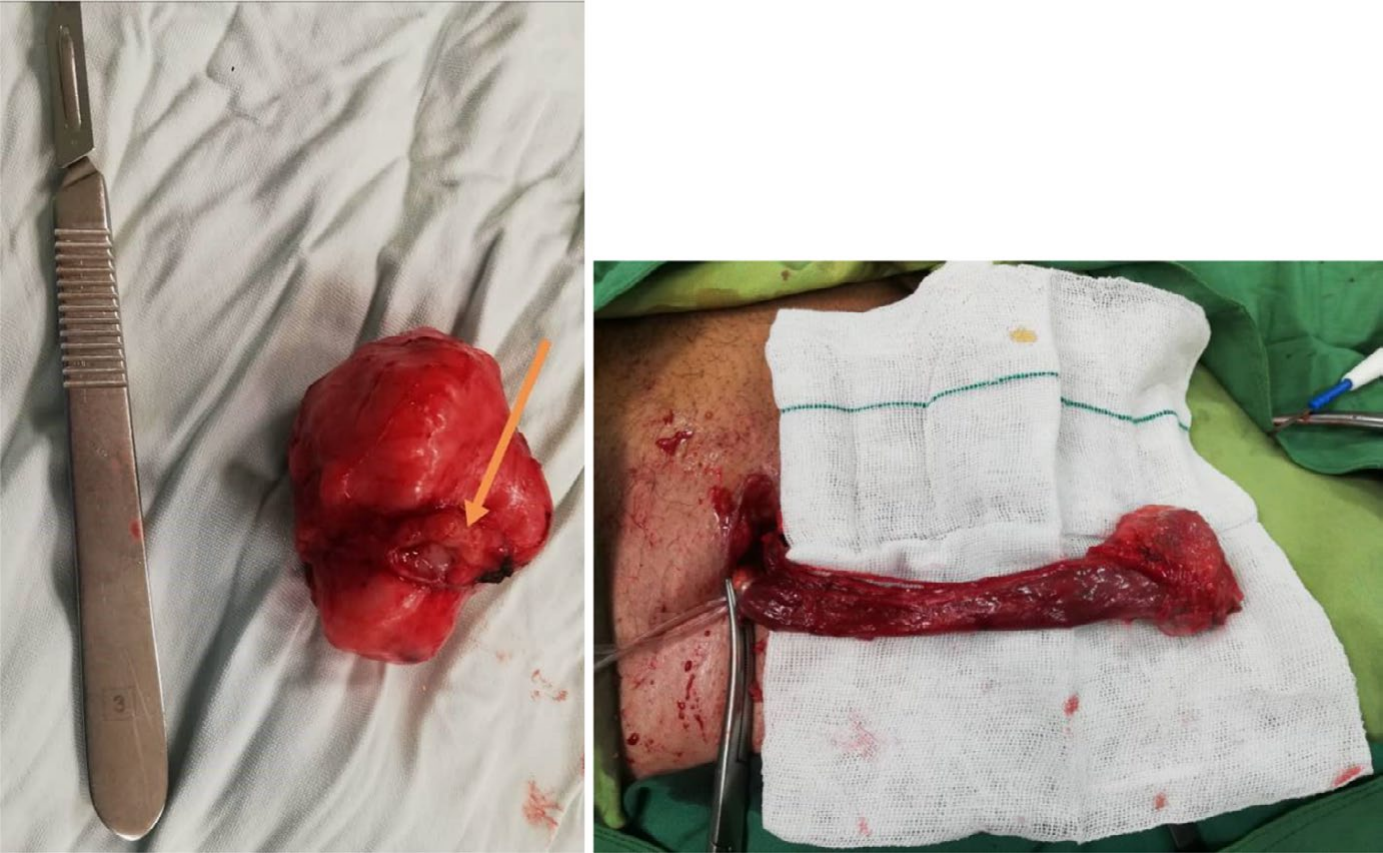
Left: Gross view of the tumor after surgical resection in the first surgery (arrow pointing at the area connected to the epididymis), Right: inguinal radical orchiectomy and high ligation of the spermatic cord in process (second surgery)

**FIGURE 2:**
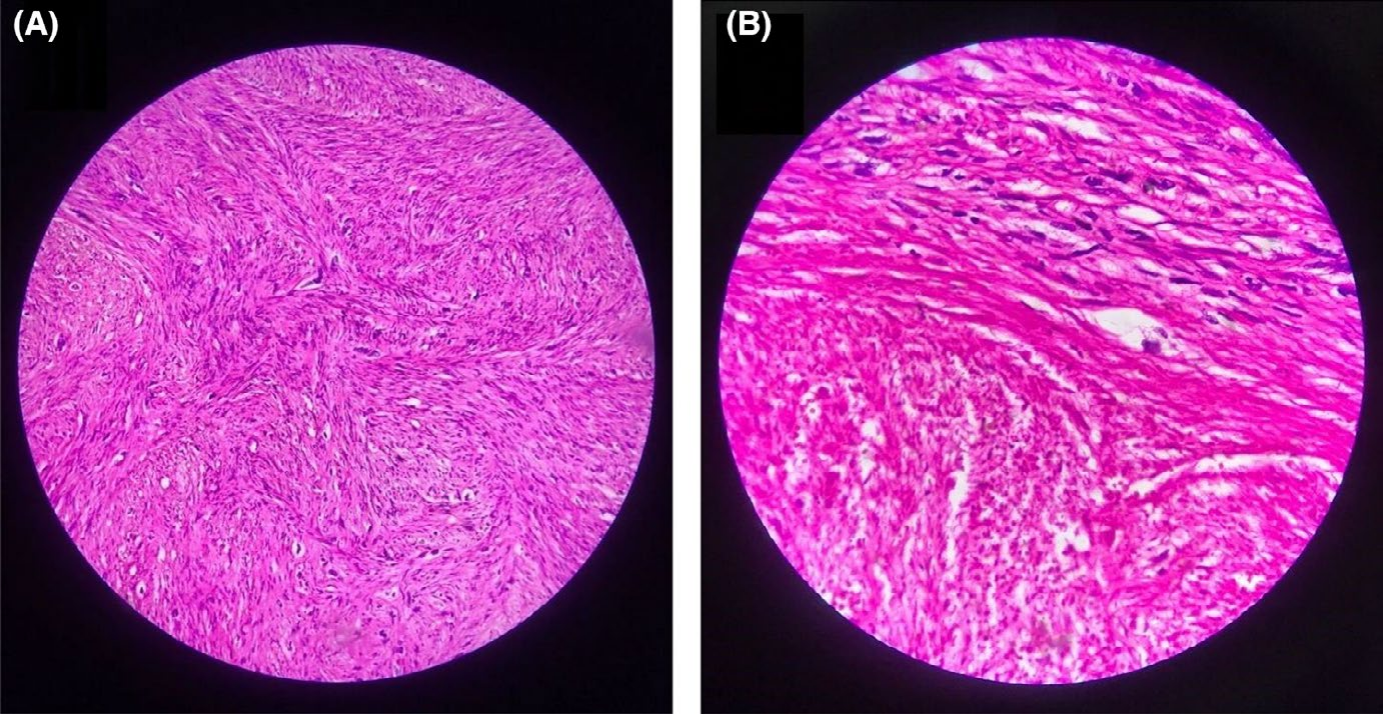
Histopathology demonstrated via hematoxylin and eosin staining. (A) low‐power field, (B) high‐power field

**FIGURE 3:**
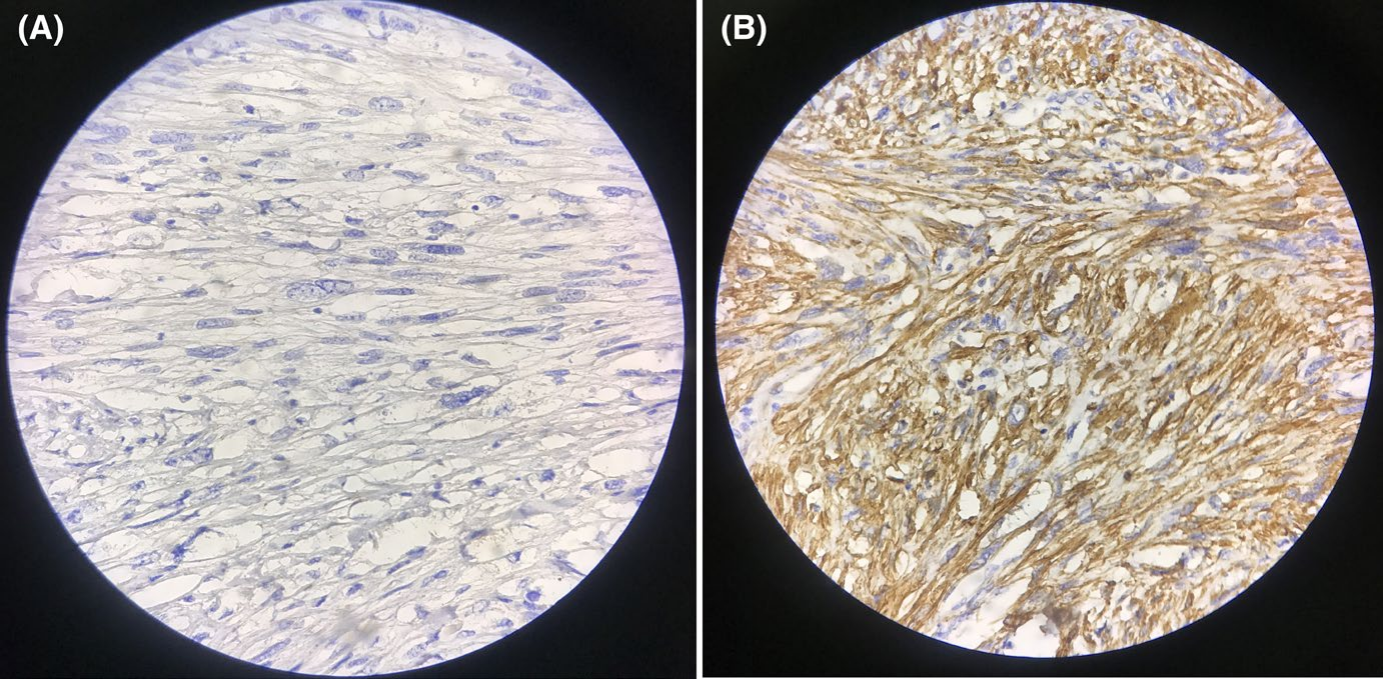
Immunohistochemistry staining which was negative for CK (A) and positive for Desmin antibody (B)

The patient underwent a scrotal surgery under general anesthesia. Intraoperative findings consisted of a para testicular firm round lobulated mass with elastic consistency measuring 5.5 × 4 cm with no adhesion to the testicle itself (Figure 1). The mass was excised and sent for pathological examination. The macroscopic view was reported as a cream‐colored lobulated 5.5 × 4 × 3 cm mass weighing 38 g. Cut sections revealed a whitish raw‐silk appearance. Microscopic description noted intersecting bundles of spindle shaped cells with eosinophilic cytoplasm and cigar‐shaped nuclei with scattered mitotic activity and moderate to severe nuclear atypia, along with regions of tumoral necrosis, compatible with the diagnosis of leiomyosarcoma, grade II. The margin at the junction of the tumor to the epididymis was less than 0.1 mm (Figure 2). Immunohistochemistry staining was negative for Cytokeratins (CK) and positive for Desmin antibody (Figure 3).

According to the histological diagnosis, reoperation was performed in 2 weeks (upon receipt of pathology report), in which inguinal radical orchiectomy and high ligation of the spermatic cord was accomplished. Testicular tissue was normal on histopathological investigation, with no tumoral involvement and vas deferens margin free of malignancy (Figure 1, right).

Further options, including adjuvant radiotherapy to reduce the loco‐regional recurrence risk, and close follow‐up for early detection of recurrence, were discussed with the patient. Nevertheless, the patient did not accept any further intervention. Although no adjuvant treatment was performed, the patient has been disease free during the last 10 months, and there is currently no clinical or para clinical evidence of recurrence.

## 3 DISCUSSION

Para testicular region contains different elements histologically, including epithelial, mesothelial, and mesenchymal tissues, which give rise to a few rare but histologically diverse tumors,^1^ with different behavioral patterns and diverse biology.^2^, ^3^

Determining the interrelation between the para testicular mass and the testicle, and differentiation between benign and malignant masses using is difficult, both in clinical examination (a painless, slow growing scrotal tumor) and radiologic features (ultrasound imaging). Histologic examination of a surgically resected specimen and morphological and immunohistochemical evaluation is needed for definitive diagnosis. In the present case, the first surgery was undertaken via a scrotal approach, assuming the presence of a benign tumor which led to the histopathological diagnosis of a LMS with a close margin, resulting in the need for an oncologic surgery.

Ünlu et al, reported and discussed the features and outcomes of seven cases of para testicular sarcoma in 2014, two of which were LMS. Both patients underwent radical orchiectomy with high cord ligation followed by adjuvant chemo‐radiation. One of the two faced mortality after lung metastasis and the other survived during the 18 months follow‐up, despite a loco‐regional recurrence.^4^ The epididymal form of LMS is less frequent than other para testicular regions. It presents as a painless, slow growing scrotal tumor like other para testicular masses, most commonly in middle‐aged or older men. It may result in the patient's discomfort because of pressure or disturbance on other nearby structures.^5^, ^6^, ^7^ On ultrasound, which is the first imaging approach in any scrotal tumor, the tumor can be a homogeneous hypoechoic lesion (similar to the present case), or have a heterogeneous pattern with areas of high vascularization.^5^ The lymphatic drainage pathway is through inguinal, external, and internal iliac nodes, which should be taken into consideration both on clinical examination and the interpretation of the CT‐scan. Like the LMS in other regions, other patterns of spread include local invasion to anatomical proximities, and hematogenous metastasis, most commonly to lung and liver. Metastatic work‐up includes thorax, abdomen, and pelvic CT‐scan. Magnetic resonance imaging (MRI) and PET‐CT‐scan are useful in detecting local and nodal invasions. Serum tumor markers and immunohistochemistry staining of the specimen can help rule out other diagnoses.^6^ In the present case, metastatic work‐up included whole body CT‐scan and serum markers, which were all performed before surgery, and all were normal.

No standard treatment protocol has been established because of the rarity of epididymal LMS. Current consensus is to perform radical orchiectomy with high ligation of the spermatic cord. If scrotal invasion is present, hemiscrotectomy is indicated.^8^ Kamitani et al, 2022, performed a retrospective analysis of 217 reported cases of para testicular LMS. Patients treated by simple tumorectomy were reported to have a significantly higher risk of a positive surgical margin (9 of 17 vs. 5 of 27, *p* = .024), which they described to be an independent risk factor for local recurrence. However, there was no significant difference in terms of DM and DSS between simple tumorectomy and high inguinal orchiectomy.^9^ Given that the presence of a positive margin leads to reoperation,^9^ it is important to adopt an appropriate surgical approach from the beginning if this diagnosis is suspected. In the present case, close epididymal margin and the final diagnosis of LMS, led to the decision for reoperation.

Considering the high risk of loco‐regional recurrence, adjuvant radiotherapy has been proposed as a useful option.^1^ There is no recent evidential study on the effect of this modality on disease recurrence or patient survival, but previous studies have mentioned this option as a rational approach, considering the fact that wide resection of all the margins are difficult to achieve and the prevalence of loco‐regional recurrence after definite surgery is 30%–50%.^10^ Rezvani et al^8^ mentioned two historical case series that described reduced loco‐regional recurrence after adjuvant radiotherapy. In addition, authors of some previous studies, recommend that adjuvant radiotherapy should be done for all types and grades of para testicular LMS, because they observed higher local failure patterns post‐surgery.^11^, ^12^ However, more studies are needed in order to take evidence based approaches, a fact also mentioned in the recent case report, presenting a patient who underwent volumetric arc conformal radiotherapy, as the adjuvant treatment for para testicular sarcoma.^13^ Although chemotherapy was occasionally used, it has no role as adjuvant treatment.^1^, ^8^ In the present case, the patient did not consent to adjuvant treatment and was followed up.

Loco‐regional recurrence patterns which are reported in the literature include the following: scrotal,^14^, ^15^ inguinal and retro peritoneal,^13^ and even gastrointestinal mucosal metastases.^16^ Despite not treating adjuvant, fortunately, the present patient did not recur or metastasize during the follow‐up period. Bhatt et al published a contemporary analysis of epididymal tumors using the US national database of 18 regions across the United States, in 2021. A total of 66 malignant epididymal tumor cases were reported between 1975 and 2016. The reported 5‐year overall survival and cancer‐specific survival rates were 84.9% and 91%, respectively. LMS was the second most frequent histopathology (second to rhabdomyosarcoma) and the leading cause of mortality (25% of cancer‐specific deaths), with a mean follow‐up of 128.6 months.^17^

The experiences above, and other documents and literature, strongly support the role of long term follow‐up for all the patients, and the need for further investigations about the role of adjuvant radiotherapy.

## 4 CONCLUSION

Soft tissue sarcoma of the epididymis must be suspected in any para testicular swelling. Because the differentiation between benign and malignant masses using is difficult, both in clinical examination and radiologic features, if there is no loco‐regional invasion or metastatic disease and the plan is surgical excision, inguinal approach is a more reasonable and safe way. After histopathological diagnosis of LMS, recommendation of adjuvant therapy and long term follow‐up is mandatory. There is a need for further investigation about the role of adjuvant treatment, considering the loco‐regional recurrence risk.

## CONFLICT OF INTEREST

The authors declare that there is no conflict of interest to be reported.

## AUTHOR CONTRIBUTIONS

M.D. involved in study concept and design. M.D. and A.K. involved in acquisition of data. M.A. involved in histopathological review and preparation of article images. M.A. and M.D. involved in drafting of the manuscript.

## ETHICAL APPROVAL

Informed consent was obtained from the patient to report their case, and the manuscript was approved at the Ethics Committee of Neyshabur University of Medical Sciences.

## CONSENT

Written informed consent was obtained from the patient to publish this report in accordance with the journal's patient consent policy.

## Data Availability

All data generated during this study can be accessed through direct communication with the corresponding author and the agreement of all research team members.
